# Neuroendocrine carcinoma of the nasal cavity with epiphora as the first symptom: A case report

**DOI:** 10.1097/MD.0000000000023502

**Published:** 2020-12-04

**Authors:** Weiqi Wu, Puying Gan, Qihua Xu, Yaohua Wang, Hongfei Liao

**Affiliations:** a Department of Ocular Oncology and Ocular Trauma, Affiliated Eye Hospital of Nanchang University, Jiangxi Research Institute of Ophthalmology and Visual Sciences, Key Laboratory of Ophthalmology of Jiangxi province; b Medical Department of Graduate School, Nanchang University, Nanchang, Jiangxi, China.

**Keywords:** CT, nasal cavity, neuroendocrine carcinomas

## Abstract

**Rationale:**

Neuroendocrine carcinomas (NECs) are rare malignancies that originate from the hormone-producing cells of the neuroendocrine system. They can grow in most organs of the body but are commonly found in the gastrointestinal and respiratory tracts. The nasal cavity is a rare site for NECs.

**Patient concerns:**

We report a case of NECs in a 45-year-old woman who presented with epiphora in the right eye for a year owing to an unknown reason.

**Diagnoses:**

The diagnosis was initially confirmed via histological and immunohistochemical assays. Postoperatively, computed tomography of the neck revealed C4 vertebral bone metastasis.

**Interventions:**

The tumor was endoscopically removed from the right eye. The patient received a full course of adjuvant polychemotherapy.

**Outcomes:**

Six months after diagnosis, the patient died due to bone metastasis.

**Lessons:**

Diagnosing nasal neuroendocrine carcinoma is clinically challenging. It must be distinguished from other orbital masses, such as chronic dacryocystitis or nasal polyps. The treatment should be further investigated for this rare malignancy in near future.

## 1 Introduction

Neuroendocrine carcinomas (NECs) are derived from neuroepithelial cells that secrete amine and peptide hormone following decarboxylation of amine precursors.^[1]^ The presence of NECs in the nasal cavity is relatively uncommon and was first reported by Dr Raychowdhuri in 1965.^[2]^

Currently, less than 100 cases have been reported.^[3]^ Owing to the complexity of its histopathological diagnosis, the pathological mechanisms of NECs remain elusive.^[4]^ NECs in the nasal cavity display aggressive local invasiveness and have a high recurrence rate.^[5]^ NECs usually have adjacent tissue invasion or distant metastasis with poor prognosis because of adjacent tissue invasion or distant metastasis.^[6]^ Clinical symptoms of this disease include rhinological syndrome and ophthalmic signs, but these are nonspecific. Surgical excision, multiple-agent chemotherapy, and radiation therapy are often utilized for its treatment. We report a rare case of nasal neuroendocrine carcinoma that primarily manifested as epiphora, including its imaging features and pathological evidence.

## 2 Case presentation

A 45-year-old woman was admitted to our institution because of epiphora in the right eye. This was accompanied by pain when she woke up in the morning. The symptoms lasted for 1 year. Her symptoms started 2 months ago when she experienced progressive right nasal obstruction. Symptoms such as bleeding, blurred vision, diplopia, nausea, and dizziness were not observed. The patient did not experience any pathological neurologic changes. No cervical lymphadenopathy was noted. Normal visual acuity and ocular mobility in both the left and right eyes were observed on ophthalmologic examination. The patient provided informed consent for the publication of this case.

Physical examination and biochemical laboratory test results, such as a complete blood count and serum chemistry panel, were within the normal range. Definitive lesions were not identified on plain chest radiography. A flaky soft tissue shadow in the right nasal cavity and lachrymal sac was noted after injection of contrast through sinonasal computed tomography (CT). Through direct nasopharyngoscopy, an exophytic fleshy mass (approximately 3 × 4 cm) was observed in the right nasal cavity. Combining both clinical and imaging features, the presumed diagnosis was chronic dacryocystitis or nasal polyp on the right side. The tumor was surgically removed through a minimally invasive endoscopic strategy with a lachrymal sac nasal anastomosis in the right eye.

**Figure 1:**
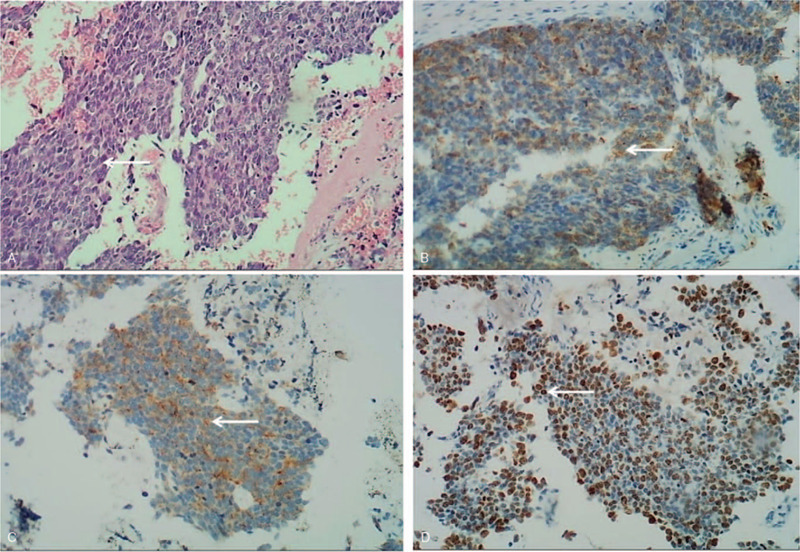
Hematoxylin and eosin staining and immunohistochemistry staining of Neuroendocrine carcinoma samples. A, Hematoxylin and eosin staining of the tumor showing large, deeply stained allodapic medium- to large-sized cells, and a scant cytoplasm (original magnification ×100). B, Positive immunohistochemistry staining for pan-CK (original magnification ×100). C, Positive immunohistochemistry staining for Syn (original magnification ×100). D, Positive immunohistochemistry staining for Ki-67 (approximately 60%) (original magnification ×100). CK = cytokeratin, Syn = synaptophysin.

**Figure 2:**
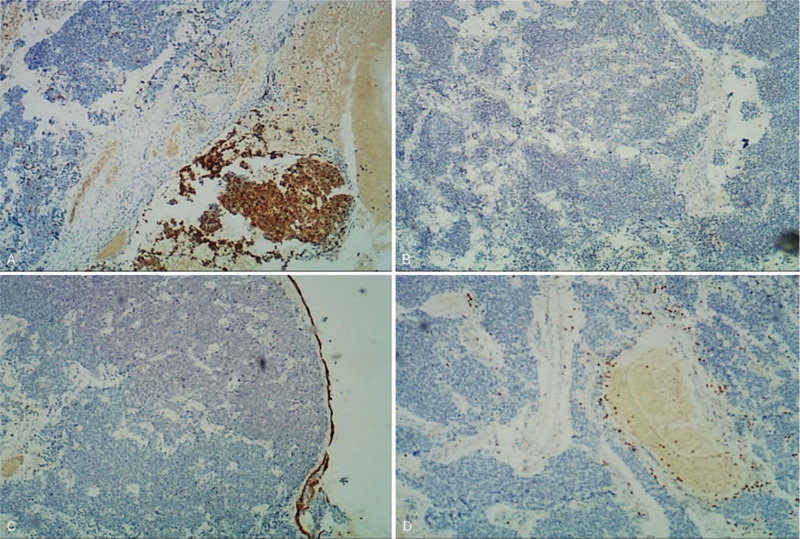
Immunohistochemistry staining for CgA (A), CD56 (B), CK5/6 (C), LCA (D) were undetectable (original magnification ×40). CgA = chromogranin A.

**Figure 3:**
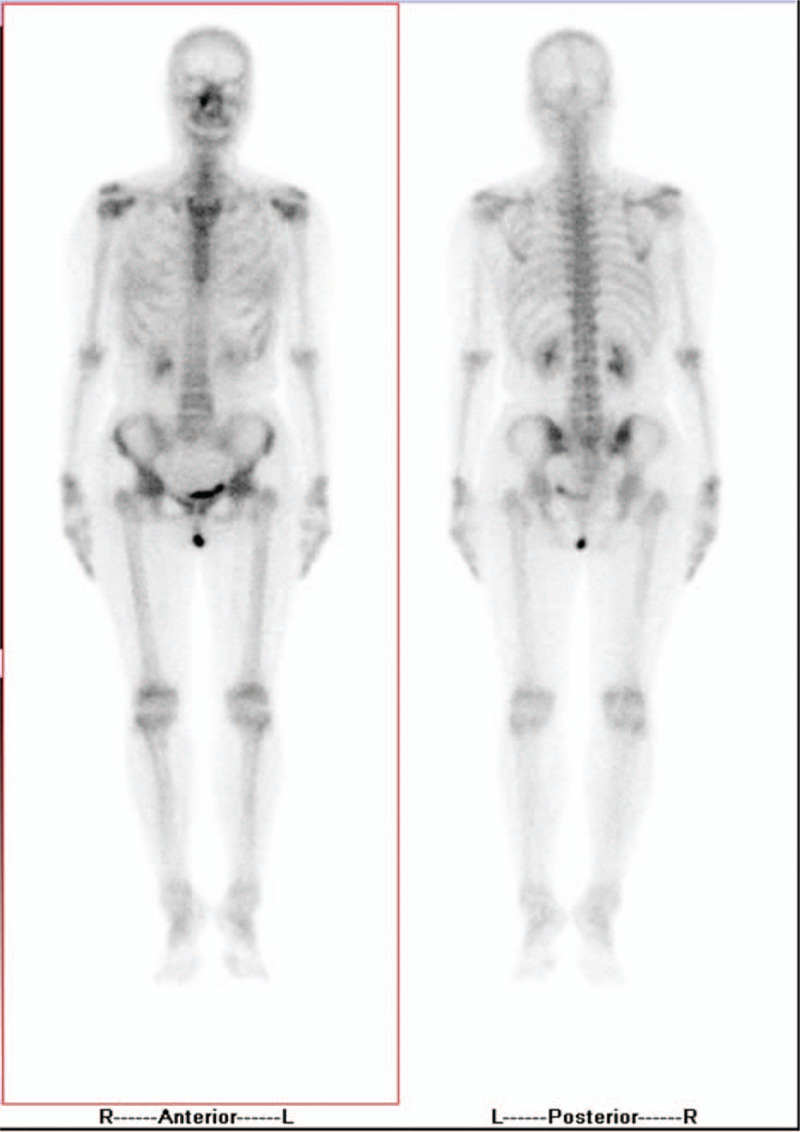
Single-photon emission computed tomography images obtained 2 h after using 99mTc MDP. Posterior whole-body images showing the abnormal nuclide concentration in the C4 vertebra. L = left, R = right.

**Figure 4:**
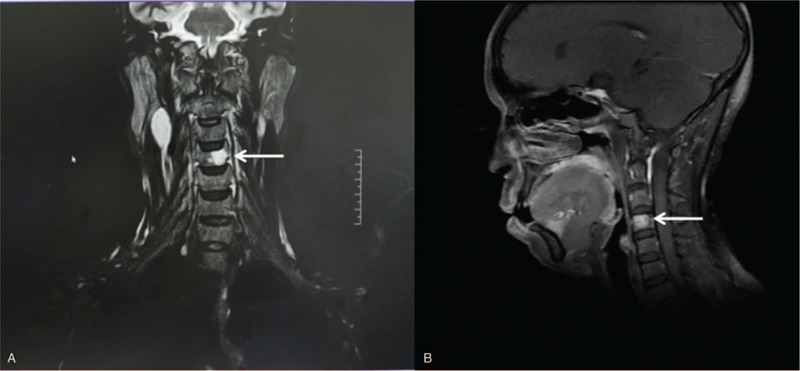
Neck magnetic resonance imaging (MRI). A, Neck MRI showing multiple and variously sized nodules in the right submandibular area with a slightly long signal on T1 or T2 and a hyperintense signal on DWI. B, Neck MRI showing the left margin of the C4 vertebral bone with a slightly long signal on T1 or T2 with bone destruction.

Pathologically, the nuclei of dysplasia cells were large and stained dark. Cytoplasmic staining was scanty (Fig. 1A). Tumor cells were positively stained with synaptophysin (Syn) and pan-cytokeratin (CK) antibodies during immunohistochemical analysis (Fig. 1B, C), which ruled out the previous diagnosis of chronic dacryocystitis or nasal polyp. The ratio of Ki-67-positive staining was approximately 60% (Fig. 1D). However, expressions of neuron-specific enolase (NSE), chromogranin A (CgA), CD56, P40, CK5/6, P63, and LCA were undetectable using immunohistochemistry staining (Fig. 2A–D). Total body bone scintigraphy and single-photon emission-CT were performed, and the results showed that the C4 vertebra had an abnormal nuclide concentration. These results indicated that vertebral bone metastasis was present in C4 (Fig. 3). Chest CT showed a shadow area in the right inferior lobe and right oblique millet. Various sizes of nodular lymph nodes in the right submandibular were found on neck magnetic resonance imaging (MRI). The size of the largest nodule was approximately 3.1 cm × 2.4 cm × 1.6 cm, with a slight long signal on T1 or T2 and hyperintense signal on DWI (Fig. 4A). The left margin of the C4 vertebral bone showed a slightly long signal on T1 or T2 with bone destruction (Fig. 4B). A combination of postoperative antineoplastic therapy and polychemotherapy with cisplatin, etoposide, and docetaxel was used for 2 periods (3 weeks per period). The patient received intensive modulated radiotherapy technique at a total dosage of 50 Gy/22 Fx within 37 days for the primary lesions and 10 Gy/5 Fx for the metastatic foci to minimize tumor growth. After 2 rounds of treatment, stage IV marrow depression occurred. Unfortunately, the patient died after 6 months of treatment.

## 3 Discussion

The World Health Organization (WHO) 2017 Classification of Head and Neck Tumors (“Blue Book”) included a new classification of neuroendocrine carcinomas (NEC) of head and neck, neck, according to the degree of malignancy.^[7]^ The NECs were divided into 3 stages, namely well-, moderate-, and poorly differentiated NECs. The poorly differentiated neuroendocrine carcinomas were further divided into small-cell NEC and large-cell NEC (LCNEC). Clinical presentations of primary NECs of the nasal cavity can lead to nonspecific symptoms such as nasal obstruction, epistaxis, or chronic sinusitis. On nasal endoscopy, the tumor mass may be seen with the naked eye as gray-white or reddish, polypoid, brittle, and it may easily bleed upon contact.^[8]^ Mass shadows can be found via CT examination of the upper nasal cavity. This is involved in the ethmoidal sinus and maxillary sinus with bone destruction. Uniform hyperintense signal on T1WI and T2WI was indicated in the enhanced MRI.

It is important to determine the extent of local tumor invasion and distant metastasis through radiological examination. CT and MRI scanning were particularly indispensable for identifying primary NECs. Zhu et al^[9]^ reported a “pigeon” pattern in the bilateral ethmoidal sinus as specific MRI features in SCNEC tumors. CT or MRI imaging of the nasal cavity has more advantages than conventional radiography when evaluating the extent of local invasion of a neoplasm. These examinations will benefit the planning of further treatment. CT has several advantages in evaluating skeletal changes. MRI complements CT because it effectively characterizes soft tissue components and evaluates the extent of tumor invasion beyond the wall of the bone sinus.^[10]^ However, the images of NECs lacked typical symptoms and special detection methods, which resulted in misdiagnosis of sinusitis or nasal polyps.^[11]^ Therefore, it is important to use CT/MRI or enhanced CT/MRI in all cases of tumors in the nasal cavity prior to surgery. Frozen pathological sections during surgery are necessary to distinguish benign and malignant tumors.

Hematoxylin and eosin staining was used to observe histopathological changes that appeared as nests, cords, and trabeculae in neoplastic cells with small cytoplasm, nucleolar deficiency, and active mitosis. For immunohistochemical staining, the neuroendocrine markers were positive for NSE, Syn, and CgA while the epithelial markers were significantly positive for CK.^[12,13]^ Generally, it is recommended to use at least 2 or more staining results combined with hematoxylin and eosin staining by synthetic analysis to determine the diagnosis of NECS.

Due to the rarity of NECs of the nasal cavity, most patients with the disease were diagnosed at later stages and developed metastasis to the liver, lung, bone, and brain.^[14,15]^ The American Joint Committee on Cancer TNM (tumor extent; the addition of extranodal extension to lymph nodes; and metastasis) staging was reported to be a predictive factor for the survival of NECs patients. Our patient had a C4 vertebral bone metastasis with T3N1M1 stage IV. The prognosis of the current case was advanced. Although no definite treatment for NECs has been established, combined surgery, chemotherapy, and radiotherapy was the primary treatment option that provided a high cure rate,^[16]^ despite the low survival.

The side effects of chemotherapy included gastrointestinal damage, bone marrow suppression, and liver damage. In our patient, we excised the tumor with a minimally invasive nasal endoscope and a 2-course cisplatin-based postoperative polychemotherapy. This induced stage IV bone marrow suppression, and the patient eventually died of bone metastasis. In a systematic review of 80 SCNEC patients studied by Rivero,^[17]^ 70% of patients presented with American Joint Committee on Cancer stage IV disease, and 37 patients (46.3%) were alive after a mean follow-up of 30.8 months (median, 15.5 months). The most common treatment modality used in 26.3% patients was combination chemotherapy and radiation therapy.

However, the second and third most common treatment modalities were combination surgery and chemoradiotherapy (21.3%), and surgery alone (18.8%), respectively. At present, the most common treatment for NECs is multimodality therapy. Previous reports showed that the local recurrence rate of SCNEC was 33%, and that of distant metastasis rate was 31%.^[18]^ It was documented that the 1-year survival rate was 57%, and the 5-year survival rate was 10%. For many patients, early diagnosis and intervention improved the outcome.

## 4 Conclusion

Neuroendocrine carcinoma of the nasal cavity is a rare but aggressive disease. Current treatment with a standard of multimodality approach should be considered for the management of NECs. However, considering the limited methods for diagnosing the disease, a multicenter study is necessary in the future.

## Acknowledgments

The authors thank Editage (www.editage.com) for English-language editing.

## Author contributions

**Conceptualization:** hong fei liao.

**Data curation:** hong fei liao.

**Formal analysis:** Weiqi Wu.

**Methodology:** Yaohua Wang.

**Project administration:** Puying Gan.

**Resources:** Weiqi Wu, Puying Gan.

**Validation:** Qihua Xu.

**Visualization:** Qihua Xu.

**Writing – original draft:** Weiqi Wu.

**Writing – review & editing:** Weiqi Wu.
